# Hygiene of the Teeth

**Published:** 1897-07

**Authors:** F. M. Bacon

**Affiliations:** Port Huron, Michigan


### THE DENTAL REGISTER.

VOL. LI.] JULY, 1897. [No. 7.

### COMMUNICATIONS.

### Hygiene of the Teeth.

BY F. M. BACON, D.D.S., PORT HURON, MICHIGAN.

It is only within recent years, that the study of dentistry has become so general, people have awakened to the fact that the care of the teeth is an absolute necessity, for it is only by the careful study of tooth development that the full truth is demonstrated.

Giving this as a mere hypotheses, if people demand proof, they will be gratified by a visit to the histologist. For by the aid of the microscope nature's unerring laws are nowhere better exemplified than in the various tissue formations and changes in the developing tooth.

This tissue which forms a future tooth consists of two classes. The first class is the stratified pavement epithelium so called because of its peculiar appearance under the microscope which shows the cells arranged in strata or layers. The second class is simply a mucous membrane found in the primary gowths. Very early along the border of the jaw appears the primitive dental groove which is caused by the membrane of the second class forming a depression in the epithelial layer. The epithelium pushes up into the membrane hardening as the process advances, and is finally what forms between the two kinds of tissue the pulp cavity, dentine, enamel, etc. When the decidious teeth are fully developed the germs of the permanent set lie embedded in the hardened tissues, awaiting the time for their growths which takes place in the same manner as does the growth of the first teeth just described. Having a brief knowledge of the early life of the tooth we are ready to more fully appreciate why such regard must be paid to the decidious or milk teeth.

Between the first teeth in the developed condition and the germs of the second, there exists such close relationship, that the nutrition and in short, the very life of the future tooth depends upon the kind of sustenance used for the maintenance of the first set.

With this knowledge we are now prepared to study and practice in detail the actual hygiene of the teeth; for what can the most difficult operations avail, if the patient neglects that most important of hygienic laws, namely: the most scrupulous care of the most important organs of our body.

It is the duty of every dentist to insist upon the strictest attention to this valuable adjunct in preserving the teeth from the ravages of decay. And now for some of the abuses and uses of this law.

The wooden tooth-pick, of such universal prestige, is regarded by many in the profession as an abomination. In probing about the proximal surfaces of the teeth, which is undoubtedly the most difficult place to cleanse, we are sure to injure and to lacerate the delicate gum tissue, thus setting up more or less of an irritation. Then, too, points and splints of the slender pick are surely broken off and are thus lodged in secure lurking places, often terminating in a severe case of periodontitis, and frequently the loosening of the tooth itself.

The dentist is oftentimes severely censured for like results from leaving ligatures upon teeth, but if a patient, with full knowledge of the evils and results of such procedure as the wooden pick, will insist upon its use, the dentist can return the censure with perfect propriety. But the patient asks "what shall we use?" The best thing to recommend is the silk floss, this does not tend to drive the foreign material into the soft tissue, but rather lifts the substance out and in no manner does it harm the gums. One may indulge in its use after each meal and especially upon retiring, always giving the lateral or drawing motion, as well as directly up and down between the teeth. The importance of this operation is seen especially since the newer and probably the correct view of the nature of dental *caries* has been under discussion. Food becomes lodged between the teeth, this contains no doubt some carbo-hydrate material which furnishes excellent media for the growth of the organisms which are incident to decay. As these micro-organisms get in their work, acids are produced which destroy the inorganic portion of the tooth. A liquifying germ soon follows which destroys the organic material or the life of that portion of the tooth affected. Hence, how essential it is, that every pocket and space between the teeth be thoroughly cleansed and as nearly *aseptic* as possible. If a probe of any kind is actually called for the pick of quill or gold is best, there being less danger in the use of these instruments of causing irritation, and no danger whatever of small pieces being lodged and retained in the tissues to cause local trouble. The next important point to be observed, in tooth cleaning, is, the proper selection and use of a brush. The uses of the brush are to remove sedimentary matters from about the teeth, to take away the partially inspissated mucous from their necks and proximal surfaces, and to impart to them a polished surface which becomes favorable to the easy circulation of the fluids of the mouth over and between the teeth. Then frequently we will have an acidulous secretion as the result of a general impairment of the functions of the alimentary canal.

With this condition we may also have a thick and ropy saliva. All this tends to the retention of foreign materials. The proper use of the brush however, diminishes to a good degree this trouble. The brush also stimulates to a more healthful action the palate gums, lips and the cheeks by a mechanical means.

The manner of using the brush is liable to so much abuse that constant instruction should be carefully given. The prophylactic is undoubtedly the most efficient of any brush on the market.

In order to produce the best results, the brush should be so made that it will reach the spaces between the teeth, and as it is to be used in a vertical direction, its curves should correspond to the curves of the arch as nearly as possible.

In prescribing tooth powders the greatest care should be used and only those who have the best knowledge of the conditions and how to treat them, should attempt to make use of such remedies which by improper manipulation would render to the part a permanent injury. A good powder assists in breaking up the film of mucous and to maintain the polished surface of the teeth by a mechanical means. It may act chemically by the action of ant-acids such as potassium or soda. It may also prevent in a marked degree the rapid growth of bacteria by the presence of some good antiseptic.

We can scarcely leave the subject of hygienic treatment of the teeth without referring to the means nature has provided conducive to their preservation. Their uses are many—the maxilla, and in fact all the dental structures are excited to a better and fuller development by the constant action of the dental organs in mastication. The food is by this process prepared for the second stage of digestion and is mixed with the normal saliva in the friction by which cleanliness is assured. That the first stage of digestion—the admixture of food with the saliva—is dependent upon a sound set of teeth, is wholly unknown to many and woefully ignored by those who are cognizant of this physiological truth. There are many persons among the ignorant and neglectful, suffering from melancholy, despondency, dyspepsia and over-strained nervous systems, brought on by malnutrition because they would not consent to visit a dentist. The excuse given by this class of individuals is fear of the operation. They were too nervous just then! or any plea which will delay the dreaded visit.

If these operations were performed while caries and other pathological conditions were only beginning, there could be nothing less painful along this line of surgery, except in some cases where a peculiar nervous organization will respond to the slightest approach upon the teeth. Shame upon the person who has not enough moral nor physical courage to meet the obligation to his Maker, by attending to these organs given him for aid in such important purposes.

One of the crying needs of the hour is attention to the first four permanent molars which take their places back of the temporary teeth. The term "six year molars" is often applied to them because it is about this period of life they appear. Many cases are brought under daily observation in which these teeth are mistaken for temporary ones and allowed to decay until irreparable injury has been done by the time dental advice is sought. If, however, the first signs of decay are properly removed and the cavities filled with suitable material, these teeth will stand the longest and strongest in the jaws. Periodical visits to the practitioner are more necessary at this time than at almost any other, because of the pre-disposition to decay. It is harder to keep the mouth in a sanitary condition than later in life; the general economy is not yet fully developed; and parents mistaking these first four permanent teeth for temporaries, lay the foundation for premature decay. It behooves all persons having young children under their charge to familarize themselves with the periods at which the permanent teeth appear. The permanent set contains thirty-two teeth, and the order of their eruption is about as follows:

| | | | | |
|---|---|---|---|---|
| First molars, . - - | 5th | to | 6th | year, |
| Central incisors, - - | 6th | " | 8th | " |
| Lateral, - - - - | 7th | " | 9th | " |
| First bicuspids, - - | 9th | " | 10th | " |
| Second " - - | 10th | " | 12th | " |
| Cuspids, - - - - | 11th | " | 12th | " |
| Second molars, - - | 12th | " | 13th | " |
| Third molars, (wisdom teeth), | 16th | " | 60th | " |

Caries is not the only way by which the teeth are lost. Before the age of 25 years decay undoubtedly holds first place; but after that time diseased gums claim quite as large a number of victims. Diseased gums are caused by poorly fitting plates, ligatures, stiff tooth brushes, careless use of pumice or charcoal, accumulated calculus, etc. After removing the cause, a soothing wash will in most cases prove a cure. If the cause is use of medicine, the physician should be notified.

The most troublesome of gum diseases to treat is pyorrhea alveolaris, meaning a flow of pus from the tooth socket. Whether from a local or systemic cause is not wholly known. Be that as it may, any collections of tartar act as irritants upon the inflammed tissues. If allowed to remain, the gum is pushed away from the neck of the tooth, causing loosening and final loss of the organ.

There is no brush which has the ability to remove the various kinds of tartar. This should emphasize the fact that the best results can only be attained by frequent visits to the dentist, who must have the hearty co-operation of the patient in every detail, if he would reach the end in view. The following, or any other good tooth powder, may be used once a day:

| | |
|---|---|
| English precipitate chalk, - - | 10 oz. |
| Finely powdered cuttle fish bone, | 6 oz. |
| " " sugar, - - - | 3 oz. |
| " " white castile soap, | 1 oz. |

A mouth wash is good when its use is indicated. Listerine and water in equal parts, makes a soothing wash and suits practical purposes. A good general mouth wash which is cleansing, healing and soothing, is the following:

| | |
|---|---|
| Chlor. potash, | ʒii |
| Acid carbolic, | ʒi |
| Glycer, - - | ℥j |
| Alcoholi, - - | ℥jii |
| Cologne, - | ʒji m |

S.—Shake and take one teaspoonful in a glass of warm water.

In connection with this, lime water should be used before retiring. Lime water or Phillip's Milk of Magnesia are also excellent in case of acid saliva. To determine as to the state of the saliva, procure a few strips of red and blue litmus paper from a druggist. Place a blue strip between the lip and gum for a few minutes; if the strip turns completely red, or is merely dotted with red spots, the saliva is acid. If a red strip turns blue, then the saliva is alkaline. If neither red nor blue are changed, the saliva is neutral.

If the saliva be acid for any length of time, the results are certainly deleterious to the teeth; hence care should be used in this direction. In connection with the above directions and hints we must not neglect the true sanitary condition of the body in general. Let there be regular hours of sleep, a regular time to eat and plenty of systematic exercise with the regular use of the bath.

It is recorded that in asylums for children of the unfortunate classes of society that as the systemic condition is improved, the teeth of the individual improve in the same ratio. Caries is diminished and oftentimes complete arrest takes place.

